# Time to abandon over-simplified surrogates of ocular perfusion pressure in glaucoma research

**DOI:** 10.1111/aos.12431

**Published:** 2014-05-19

**Authors:** Anthony P Khawaja, David P Crabb, Nomdo M Jansonius

**Affiliations:** 1 Department of Public Health and Primary Care, Institute of Public Health, University of Cambridge School of Clinical MedicineCambridge, UK; 2 Department of Optometry and Visual Science, City University LondonLondon, UK; 3 Department of Ophthalmology, University Medical Center Groningen, University of GroningenGroningen, The Netherlands; 4 Department of Epidemiology, Erasmus Medical CenterRotterdam, The Netherlands

Editor

The role of ocular perfusion pressure (OPP) in the pathogenesis of glaucoma has attracted a great deal of research interest, as highlighted in a review article recently published in *Acta Ophthalmologica* (Costa et al. 2013). For the majority of studies in this field, blood pressure (BP) minus intraocular pressure (IOP) has been used as a simple surrogate measure of OPP. The authors of the review article correctly acknowledge that interpretation of these surrogate measures is problematic and that any crude association observed between OPP and glaucoma may be related solely to the IOP component, given the known strength of IOP as a risk factor for glaucoma. However, we strongly disagree with the authors’ conclusion that statistically adjusting for IOP is a satisfactory solution to this problem. We have previously shown that it is quite impossible to untangle the effects of IOP and BP in a model containing OPP results (Khawaja et al. 2013). In short, adjusting for IOP in a model containing OPP will inevitably result in the situation that the coefficients for OPP actually represent the effect of BP only, and not OPP. This has been substantiated theoretically and demonstrated clearly using a simulated dataset; the coefficients for OPP in IOP adjusted regression models were exactly the same as those for BP in IOP adjusted models (Khawaja et al. 2013). Therefore, in studies that have found a significant association between OPP and glaucoma using regression models adjusted for IOP, it is actually a significant association between BP and glaucoma that has been demonstrated, and no conclusions can be drawn regarding perfusion pressure. OPP may well be important in glaucoma pathogenesis, but unfortunately the surrogate approach of calculating BP minus IOP is over-simplified, and results have been consistently misinterpreted, which has not furthered our understanding of the disease process. Given the critical issues highlighted, we strongly suggest abandoning the approach of assuming that BP minus IOP reflects OPP and that research resources are directed towards new methods in this important field.
